# Older Adults and Positive Mental Health during the Second and Sixth COVID-19 Waves in Spain

**DOI:** 10.3390/healthcare10112159

**Published:** 2022-10-28

**Authors:** Carmen Moret-Tatay, Alexis Cloquell-Lozano, Marcelino Pérez-Bermejo, Francisco Javier Arteaga-Moreno

**Affiliations:** 1 OAMI-UCV, Universidad Católica de Valencia San Vicente Mártir, 46001 Valencia, Spain; 2 MEB Lab, Faculty of Psychology, Universidad Católica de Valencia San Vicente Mártir, Avenida de la Ilustración 4, 46100 Valencia, Spain; 3 SONEV Research Group, School of Medicine and Health Sciences, Catholic University of Valencia, C/Quevedo nº 2, 46001 Valencia, Spain

**Keywords:** COVID-19, ageing, sex differences, positive mental health, coping

## Abstract

The spread of the COVID-19 virus was a worldwide phenomenon, which was unprecedented in modern times. The restriction measures can be perceived as a heavy burden for mental health during this period, particularly for some groups. The aim of this study is to examine a positive mental health model across ages, where a moderated mediation model is proposed involving sex differences and confidence in coping with COVID-19. Two independent samples were studied during the second and sixth waves in Spain: *n* = 2861 and *n* = 2462, respectively. The main conclusions can be described as follows: (i) while age was not related to mental health during the second wave, a positive relationship was found between it and the sixth one; (ii) age was positively related to the confidence in coping with COVID-19 during both of the waves; (iii) women showed worse scores for the variables in the study than the men did during the second wave, but this pattern was reversed in the sixth one; (iv) after a moderated mediation model on the relationship between age and positive mental health in terms of confidence in coping with COVID-19 and sex, an interaction was found for the second wave but not for the sixth one. These results suggest that older adults and women would develop more strategies and resources for a positive mental health across time.

## 1. Introduction

**Figure 1:**
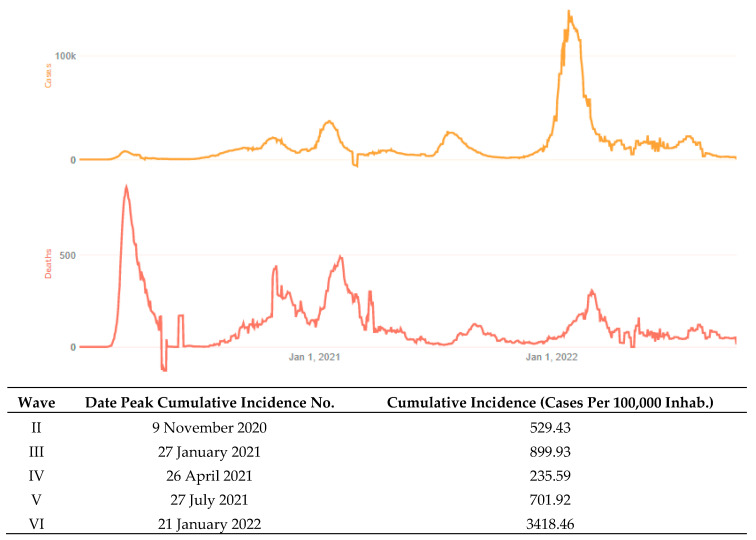
On the top: cases and death data that were adapted from JHU CSSE; testing and vaccine data from JHU CCI; hospitalization data from the U.S. Department of Health and Human Services. On the bottom: cumulative peak incidence across COVID-19 waves in Spain. Author’s own elaboration based on data that were obtained from the Red Nacional de Vigilancia Epidemiológica (RENAVE).

A COVID-19 pandemic (from Coronavirus disease 2019) was declared by the World Health Organization (WHO) in mid-March 2020. Many countries adopted several socio-health measures, such as quarantines. A state of alarm was declared throughout the Spanish territory on 14 March 2020 by Royal Decree 463/2020. In this regard, a series of measures such as lockdowns and social distancing were adopted to prevent the spread of the virus and to reinforce the National Health System. By 21 June 2020, Spain entered the so-called “new normality”. By 25 October 2020, a new state of alarm was declared. Since then, not only different waves have been experienced with different levels of restriction in the country, but also different virus mutations, such as the omicron one in the sixth wave. It should be noted that the mortality rate of the sixth wave was lower than it was in the previous waves (Center for Systems Science and Engineering at Johns Hopkins University, JHU CSSE, 2022), although the peaks of the cumulative incidence were higher, as depicted in Figure 1.

It should be also noted that the vaccine coverage might have prevented hospitalizations and more specifically ICU (intensive care unit) occupancy as Spain showed relatively lower levels of hesitancy in being vaccinated and thus, higher vaccination rates [1]. In this scenario, the restriction measures can be perceived as a heavy burden, particularly for mental health [2,3].

For most researchers, older adults have often been considered as one of the most vulnerable groups during the COVID-19 pandemic [4]. This historic milestone has exposed some fragilities of the healthcare system, evidencing the highest mortality rates in older people with comorbidities and functional impairments [5,6,7]. Nevertheless, the crisis also provided an opportunity for the development of learning solutions, including supporting more positive mental health. For example, the literature has shown how, despite the evident barriers and restrictions, older adults also seem to have shown a greater number of coping strategies when they are compared to other age groups [8]. 

The successful lockdown of the elderly leaves us with an invaluable lesson about personal strength in facing situations of fragility and vulnerability. Despite concerns that older adults would respond to COVID-19 with increased loneliness and isolation, the research shows that they had better outcomes than other groups did [9]. In a study that was carried out in Spain, this group showed better scores on both the positive and negative effects when they were compared to the younger group, as well as more frequently reported positive daily events [10]. In this regard, the literature has also claimed that older adults who had a greater confidence in coping with the disease were more likely to adopt authority-suggested preventive behaviors [11,12].

Another variable of interest is related to the sex differences that were reported during the COVID-19 pandemic [13,14]. According to a piece of research, women tended to be more vulnerable in terms of stressor exposure at a younger age and having to adopt to a caregiver status during the pandemic [15]. Moreover, they had higher scores on the affective dimension, and more precisely, regarding anxiety and fear of personal death [16,17]. The literature has presented different mediational models on mental health during the COVID-19 outbreak [16,18,19]. Considering that the literature has presented a mediated relationship between the confidence to cope and positive attitudes, as well as subjective well-being [20,21], sex differences are a subject of interest in the field. 

**Figure 2:**
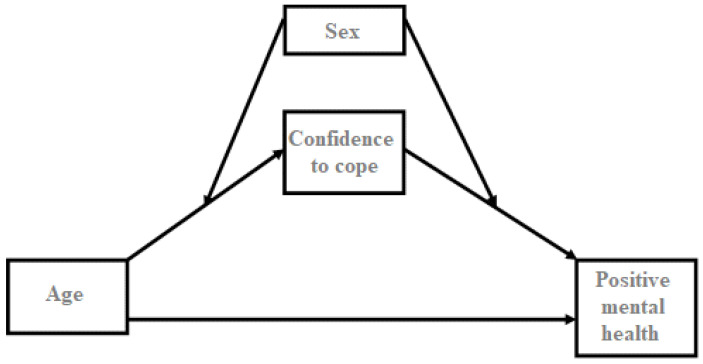
Theoretical model tested. Variables under study: age, sex, confidence on coping with COVID-19 and positive mental health.

As depicted in Figure 2, a positive mental health model is proposed in this regard. First, ageing is expected to predict the confidence to cope with COVID-19 and positive mental health scores. This prediction is based on previous studies that have claimed that there are age-related differences in stable mental health, as well as certain determinants of health, such as sex, which differ across the life-course of a person [22]. Given the previous results, it is hypothesized that age is a predictor of positive mental health, and a moderated mediation model is proposed across the aforementioned relationship, including variables such as confidence in coping with the disease and sex. 

In terms of the context, the comparison of the previous model across the different waves of the COVID-19 pandemic might shed light on the mental health impact that it had, particularly in older adults and the sex-related differences in this. In this way, this work also aims to compare positive mental health at the beginning of the pandemic and its evolution over time.

## 2. Materials and Methods

### 2.1. Participants

Participant data were obtained from the Centro de Investigaciones Sociológicas (CIS) barometers during the second “Effects and consequences of Coronavirus. Study 3302 (November 2020)” and the sixth wave “Effects and consequences of Coronavirus. Study 3346 (December 2021)” in Spain. 

The second wave was chosen because it was the closest to the beginning of the pandemic (there are no data which are available for the first wave), while the sixth wave represents a change of context with the emergence of vaccines and the omicron variant. Thus, two independent samples were employed for two different moments. Inclusion criteria can be described as follows: (i) the participants had to be over 18 years old, and (ii) they had to be a resident in Spain. 

The sample for the first study consisted of 2861 people. A total of 51.9% of respondents reported being women. The mean age was 50.50 years (SD = 16.56), with this ranging from 18 to 90 years old. With regard to their level of educational studies, 2.9% of them had not studied, 8.1% of them studied at a primary level, 29.8% of the participants reported studying at a secondary school, 21.8% of them had professional qualifications and 37.1% of them had a higher or further education degree.

For the second sample, a total of 2462 people volunteered to participate in the study. Of the respondents, 50.2% of them reported being women. The mean age was 50.31 years (SD = 16.52), with the ages ranging from 18 to 91 years old. In this case, with regard to their level of educational studies, 3.1% of them had not studied, 7.3% of them studied at a primary level, 30.3% of the participants reported studying at a secondary school, 18.8% of them had professional qualifications, 39% of them had a higher or further education degree, and 1.5% of them reported other categories that were not included or preferred not to answer.

### 2.2. Procedure and Materials

The CIS barometers are designed to be statistically representative of Spanish society. For this reason, sampling was conducted via a multi-staged, stratified cluster procedure, and it is considered to be Spain’s most comprehensive data [23].

Variables that were under study included sociodemographic data, as well as the following questions:

(i) Second order questionnaire on coping with COVID-19 composed of 9 questions (question 3 is in the CIS study 3302, and question 2 is in the 3346 study). This questionnaire mainly involves emotional coping issues (fear, worry and dread, among others). Although the scale is dichotomous in its nature, the Cronbach’s α and McDonald’s ω coefficients were used as an approximation to KR (Kuder–Richardson) for internal consistency. In this way, Cronbach’s α was 0.571 for the 2nd wave and 0.651 for the sixth one, while McDonald’s ω was 0.575 for the 2nd wave and 0.650 for the sixth one. These measures were used as a second order method.
(ii) Likert 4-point inverted scale with 6 items for positive mental health (question 4 in both the CIS studies 3302 and 3346), containing the main question: How have you been feeling lately? In terms of internal consistency, Cronbach’s α was 0.786 for the 2nd wave and 0.777 for the sixth one, while McDonald’s ω was 0.791 for the 2nd wave and 0.781 for the sixth one.

### 2.3. Design and Analysis

This is a cross-sectional study that was conducted at two points in time during the COVID-19 pandemic in Spain (the second and sixth waves). The software SPSS version 23 (IBM) was employed. Normality and homogeneity analyses of data were conducted prior to the analyses. Data were standardized before the moderation mediated analysis, which was carried out using the Process macro for SPSS [24,25]. In this way, regression-based procedures were executed employing bootstrapping procedures using 10,000 samples.

## 3. Results

**Figure 3:**
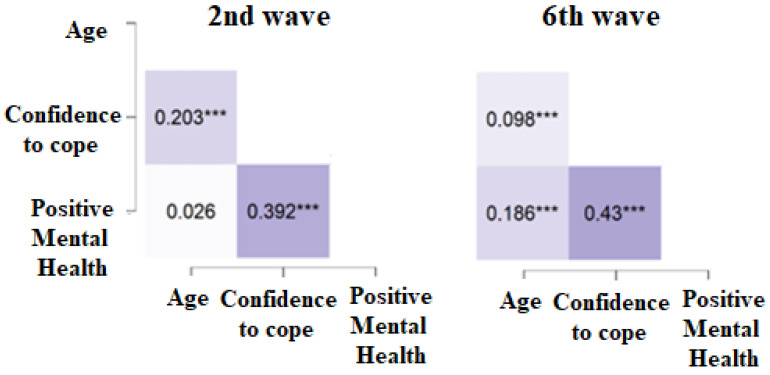
Pearson’s coefficient correlation heat map across the 2nd and 6th waves in Spain. Note; *** *p* < 0.0001.

First, the descriptive analyses were carried out across the sex variable. Table 1 depicts the descriptive approach, including the t-tests for the independent samples and its underlying effect size through Cohen’s d. The relationship between the variables of interest and age is described in Figure 3. These values were slightly different across sex, noting that there was a change in the pattern across the second and sixth waves. 

A linear multiple regression was carried out (see Table 2) for the 2nd and 6th waves. Thus, age, confidence in coping with COVID-19 and sex (considered as a dummy variable) were entered as the predictors, and the outcome variables was positive mental health.

The Adjusted R^2^ for the 2nd wave set was 0.19, and the resulting model was statistically significant; F(3,2820) = 215.99; MSE = 1943.13; *p <* 0.001. In the case of the 6th wave, the R^2^ was 0.21, and the resulting model was significant; F(3,2461) = 214.43; MSE = 1343.14; *p <* 0.001.

Lastly, two different moderated mediation models were assessed regarding the differences across the two waves in the study. Table 3 depicts the regression coefficients, as well as the 95% confidence interval (CI) that was statistically significant with a confidence interval excluding the zero value, by reporting both the lower (LLCI) and upper levels (ULCI) for all of the four analyses. It should be noted that an interaction between age, sex and confidence in coping was statistically significant for the second wave, while it did not reach the statistical level for the sixth one (*p* > 0.05).

## 4. Discussion and Conclusions

The aim of this study was to examine a moderated mediation model across the relationship between age and positive mental health. A mediation effect for the confidence in coping with COVID-19 was expected across the previous relationship, as well as a sex moderation on the named mediation. This model was carried out for two COVID-19 waves in Spain, the second and sixth ones. 

According to the literature, a better positive health status of older people has been found when they were compared to other younger groups during the COVID-19 pandemic [9,10]. The current results support the previous literature partially in the second wave by showing a positive relationship between confidence in coping with COVID-19 and age, but not for positive mental health and age. Nevertheless, this pattern changed over time. In this regard, it is hypothesized that older adults would develop more strategies and resources to adapt to the new conditions and restrictions. In other words, older adults would show a higher increase in the capacity to psychologically cope and adapt during COVID-19 over time as described in previous literature [8]. One should also bear in mind that young people, on the contrary, have had to bear the pressure of labor changes in this period [26], unlike older adults, who in many cases would not be in active employment, which is why future lines of research should address the economic, social and, above all, the labor variables [22].

Another aspect of interest is related to the differences between men and women. Not focusing on specific cases of vulnerability, such as health restrictions during pregnancy or violence against women [27,28], the previous literature seems to support the vulnerability and adverse impacts that occurred to women’s mental health during this pandemic [15,16,17,29]. According to a piece of research that was carried out in Italy from 7 to 10 weeks during the lockdown, to be a women under 45 years, working from home or being underemployed were relevant risk factors for worsening cognition and mental health [30]. A comparison of some of these factors over time was of interest in the present study. The current data would support the differences between men and women during the second wave, describing a worse situation for women in terms of their positive mental health and confidence in coping with COVID-19. However, this pattern seems to change during the sixth wave, depicting an inversion of the scores. Although this difference could be considered subtle when it is compared to that of the men, an improvement in the adaptive resources for women could be inferred. In this way, future lines of research should address adaptive resources as a moderating variable to understand its role in affecting positive mental health statuses. 

Regarding the proposed moderate mediation model, the current results support the adaptability mechanism for positive mental health during COVID-19 over time. The interaction that was found between the age and sex variables in confidence to cope during the second wave, but not during the sixth wave, would support this result. The previous literature has shown how negative emotions can be beneficial because of their adaptive effects that allow individuals to maintain their ability to cope with stressful situations, such as the COVID-19 pandemic [31]. The present results would support this hypothesis, particularly for the differences between men and women across their lifespans and their ability to cope after a stressful situation.

These results are of interest at both the theoretical and applied levels. Firstly, for the implementation of the theoretical models on mental health, particularly in relation to age and sex. Secondly, and at the applied level, for the training programs for mental health professionals. As described in previous literature, health-promoting interventions should be lifespan-sensitive [22]. In this way, these results seem to be promising with respect to the adaptive capacity of the groups that are under study, and especially in the fight against the stereotypes such as ageism [32,33] of sex-linked stereotypes [34]. The main limitations of the current study are mainly related to the design of it. On one hand, as it was a survey on the perception of the participants facing the COVID-19 outbreak, some individual biases may have occurred. On the other hand, the design of it is cross-sectional at two different points in time, whereas a longitudinal study with the same participants would have been more desirable. In any case, given the type of sampling and the representation of the Spanish population, we consider it of interest on this front.

The main conclusions can be described as follows: (i) while, age was not related to mental health during the second wave, a positive relationship was found for the sixth one; (ii) age was positively related to the confidence in coping with COVID-19 at both of the waves; (iii) women showed worse scores than men did during the second wave, but this pattern was reversed in the sixth one; (iv) after a moderated mediation model on the relationship between age and positive mental health in terms of the confidence in coping with COVID-19 and sex, an interaction was found for the second wave but not for the sixth one.

## Figures and Tables

**Table 1:** Descriptive analysis on variables of interest across sex.

		Confidence to Cope			Positive Mental Health		
Variables		Women	Men	*p* (d’)	Women	Men	*p* (d’)
2nd Wave	Mean	14.270	14.821	<0.001	18.108	19.507	<0.001
	*SD*	2.923	2.875	(−0.19)	3.466	3.003	(−0.43)
6th Wave	Mean	13.880	13.202	<0.001	19.868	18.509	<0.001
	*SD*	2.841	2.738	(0.24)	3.084	3.476	(0.41)

**Table 2:** Variables included in the linear regression model for the 2nd and the 6th waves. Sex was included as a dummy variable. Standard error = SE; Unstandardized = B and Standardized = β. Lower and upper confidence interval (LLCI and ULCI) level.

Coefficients								
							95% CI	
Model		B	SE	β	*t*	*p*	LLCI	ULCI
	(Intercept)	12.881	0.309		41.657	<0.001	12.275	13.488
2nd wave	Confidence to cope	0.477	0.022	0.386	22.128	<0.001	0.435	0.520
	Sex	1.146	0.114	0.172	10.064	<0.001	0.923	1.369
	Age	−0.009	0.004	−0.043	−2.449	0.014	−0.015	−0.002
	(Intercept)	13.540	0.057		239.133	<0.001	13.429	13.651
6th wave	Confidence to cope	0.341	0.015	0.407	22.105	<0.001	0.311	0.371
	Sex	−0.224	0.103	−0.040	−2.176	0.030	−0.426	−0.022
	Age	0.025	0.003	0.147	8.147	<0.001	0.019	0.031

**Table 3:** Direct and Conditional effect of X on Y at values of the moderator. Effects, standard error (SE), statistical significance and lower and upper (LLCI and ULCI) levels.

Model		Group Measures	Effect	*SE*	*p*	LLCI	ULCI
2nd wave	Direct effect		−0.04	0.01	<0.05	−0.07	−0.005
	Indirect effect	Men	−0.48	0.10	<0.05	0.07	0.12
		Women	0.51	0.05	<0.05	0.03	0.07
	Confidence to cope	Constant	−0.003	0.01	0.84	−0.03	0.03
		Age	0.20	0.01	<.001	0.16	0.23
		Sex	0.21	0.03	<0.01	−0.19	−0.05
		Interaction	−0.12	0.03	<0.01	−0.19	−0.05
	Positive Mental Health	Constant	0.001	0.01	0.97	−0.03	0.03
		Confidence	0.38	0.02	<0.01	0.33	0.43
		Age	−0.04	0.01	<0.05	−0.07	−0.005
		Sex	0.34	0.03	<0.01	0.27	0.41
		Interaction	−0.01	0.04	0.80	−0.11	0.08
6th wave	Direct effect		0.14	0.01	<0.01	0.11	0.18
	Indirect effect	Men	0.04	0.01	<0.05	0.02	0.07
		Women	0.03	0.01	<0.05	0.01	0.06
	Confidence to cope	Constant	0.66	0.06	<0.01	0.48	0.73
		Age	0.11	0.06	0.05	−0.03	0.24
		Sex	−0.40	0.03	<0.01	−0.48	−0.33
		Interaction	−0.01	0.03	0.75	−0.08	0.06
	Positive Mental Health	Constant	0.11	0.05	0.04	0.002	0.23
		Confidence to cope	0.44	0.06	<0.01	0.32	0.56
		Age	0.14	0.01	<0.01	0.11	0.18
		Sex	−0.07	0.03	<0.01	−0.15	−0.007
		Interaction	−0.02	0.03	0.52	−0.09	0.04

## Data Availability

Data are obtained from Centro de Investigaciones Sociológicas (CIS) barometers and freely available online. The data is available online at https://www.cis.es/cis/opencm/ES/1_encuestas/estudios/ (accessed on 19 June 2022).
